# Domotics Project Housing Block

**DOI:** 10.3390/s16050741

**Published:** 2016-05-23

**Authors:** Carlos Morón, Alejandro Payán, Alfonso García, Francisco Bosquet

**Affiliations:** Grupo de Sensores y Actuadores, Dpto. Tecnología de la Edificación, Universidad Politécnica de Madrid, 28040 Madrid, Spain; alejandro.payandetejada@gmail.com (A.P.); alfonso.garciag@upm.es (A.G.); frbosquet@gmail.com (F.B.)

**Keywords:** home automation, energy-saving, digital home

## Abstract

This document develops the study of an implementation project of a home automation system in a housing placed in the town of Galapagar, Madrid. This house, which is going to be occupied by a four-member family, consists of 67 constructed square meters distributed in lounge, kitchen, three bedrooms, bath, bathroom and terrace, this being a common arrangement in Spain. Thus, this study will allow extracting conclusions about the adequacy of the home automation in a wide percentage of housing in Spain. In this document, three house automation proposals are developed based on the requirements of the client and the different home automation levels that the Spanish House and Building Automation Association has established, besides two parallel proposals relating to the safety and the technical alarms. The mentioned proposed systems are described by means of product datasheets and descriptions, distribution plans, measurements, budgets and flow charts that describe the functioning of the system in every case. An evaluation of each system is included, based on other studies conclusions on this matter, where expected energy savings from each design, depending on the current cost of lighting, water and gas, as well as the expected economic amortization period is evaluated.

## 1. Introduction

Usually, in the building sector, it is forgotten that the right functioning of a building does not exclusively depend on its distribution, constructive features or facilities. Actually, the most important factor is the way the building is used.

In this field, domotics play a vital role, getting the most of its facilities and constructive features. The domotics concept, as home automation, develops new systems that are able to automatize common functions in households from developed countries, like electrical facilities management, audiovisual systems, *etc.* However, currently, there is another concept related to domotics, considered the next step in this area. Smart house concept, apart from home automation, includes central data control of the elements of the system [1].

Domotics is an essential part of the future of building sector, in which some new services and comforts will be integrated in households. Nowadays, this automation is becoming very important for building energy efficiency, and reducing energy consumption [2] and CO_2_ emissions. These facts are demonstrated in the study of Ippolito [3], in which the certification level is improved, according to EN15217 standard [4].

One of the cornerstones in which more efforts are being carried out, is energy efficiency, including making passive systems into active systems, regulating demand, and adapting these systems to the usage of the building [5]. In fact, in some cases, consumption peaks have been decreased by 46% [6]. There is hope that these systems will be more affordable, while maintaining their efficiency [7].

Furthermore, we should not forget that these systems produce intangible benefits for the user. Feeling protected against theft, preventing installation failures, and increased comfort level because of the control of environmental variables are added to energy efficiency benefit with the consequent economic savings. 

These systems are objects of continuous improvement, making Home Building Automation System (HBAS) or Building Automation System (BAS) more reliable and robust. In addition, model-based and interactive simulations have been developed [8]. Another example is the inclusion of wireless technologies like ZigBee or 6LoWPAN, favoring their interoperability [9].

The inclusion of more and better sensors improves the ability of the system to adapt to the environment and promote the requirements for keeping desired conditions, using a minimum of energy for that performance. Examples might include the inclusion of rain, solar radiation (direct or diffuse), and humidity or wind sensors, among others [10].

On the other hand, from the user´s point of view, many different services have been created. Internet Protocol Television (IPTV) services [11] or Android supported scalable home automation systems [12] are two examples. Moreover, some studies are focused on creating systems that help dependent people, including elderly people, such as the low-cost system by Dasios *et al*. [13] or the security and comfort system from Carpio *et al.* [14]. Other systems are capable of gathering data via the Internet from public Application Programming Interfaces (APIs) [15]. It is also important to include multi-agent platforms such as PANGEA, used in the work of Villarubia *et al*. [16].

More in general, greenhouses are a good example. In them, climatic conditions control is essential for the installation’s function and automatic devices are proving to be very useful [17].

Many technologies are used in domotics area, creating a worrying heterogeneity in this area [18]. Among the various domotics technologies, there are two manufacturers that are more representative because of their major contribution to the area, LonWorks, prevailing in North America, and Konnex (KNX) in Europe. There are a great number of works based on KNX technology that demonstrate its great utility in home data gathering [19]. Moreover, a KNX system can link the city in which it is situated to its resources management system [20].

In this work, a domotics system implementation in a flat located in Galapagar (Spain) based on KNX technology is studied [21].

To create a more detailed study, three systems have been implemented to achieve each level of home automation specified in UNE-CLC/TR 50491-6-3:2013 IN [22]. Furthermore, we also designed and installed two independent modules compatible with the above-mentioned systems, a security subsystem and a system related to technical alarms.

The aim of this work is to carry out a viability study of each KNX system level for small apartments. This viability is studied in regard to economic, environmental and energy decisions. Moreover, we have gathered data about energy savings of each system and they have been compared according to the three mentioned areas. We have also simulated with SeeTool^®^ software the results of two more real small apartments (Barcelona and A Coruña) with the same orientation located in different climate zones.

For this purpose, and after implementation of these systems in the flat, a quantitative analysis of energy and economic variables has been carried out as well as a second qualitative study made by means of satisfaction surveys that contain comfort and security questions, after the user had been living together with the systems.

Based on these two analyses, we present several conclusions about the influence of each system in user’s life and the energy and economic savings these systems have resulted in.

## 2. Materials and Methods

### 2.1. Subject of Research

We have designed and installed a home automation in a flat located in Galapagar (Spain). This flat has a north orientation and consists of 67 m^2^ usable space over a 79 m^2^ constructed area, distributed in several rooms: a living room-kitchen-hall (26.80 m^2^), a master bedroom (12.29 m^2^), two additional bedrooms (10.24 and 8.34 m^2^), a bathroom (3.72 m^2^), a toilet (1.61 m^2^), one corridor (3.40 m^2^) and a terrace (12.24 m^2^).

### 2.2. General Features of the Home Automation System

- Physical level: In this case, a TP1 wiring type for data transmission was chosen. The choice of a wired system instead of a wireless solution, which is a rising technology, is due to the fact that this flat was to be integrally rehabilitated. This makes the added costs of wiring not so high. Apart from that, wiring solutions are highly reliable and using them, we can eliminate added electromagnetic contamination [23], which has been increasing for the last years because of the uncontrolled advance of technology. It makes wiring solutions a solution to take into consideration.
- Topology: We have chosen a data bus composed of lines and areas joined by line-couplers (LC) and backbone/area couplers. Each line can host 64 devices, and each area can be formed for up to 15 lines. Each line is powered by a 24 V DC power supply, and each one supports 640 mA with a cushioning time of 100 ms.
- Network design: Each device in this system consists of a microcontroller with memory. This way, we have achieved a distributed architecture, in which if one device fails, it will not affect the functioning of the whole system. Furthermore, it facilitates possible future expansions of the system. Each device is individually programmed.
- Protocol: This system is based on KNX standard because it is an already-tested system and because of its reliability and compatibility with many products from different manufacturers. The information is symmetrically sent by both conductors as an alternating signal superimposed on a 24 V dc current. Likewise, KNX use Carrier sense multiple access with collision avoidance (CSMA/CA) network multiple access method in which carrier sensing is used, but nodes attempt to avoid collisions by only transmitting when the channel is sensed to be clear.
- Devices: Each device consists of three elements, bus coupler, which is composed of three memory chips (EPROM, ROM and RAM); an external-physical interface; and an application module.

### 2.3. Common Elements

Regardless of the home automation level, there are some elements that are common to all systems being implemented in them. The first one is the power supply; it is necessary to generate the needed voltage in each line. The power supplies we have chosen are suitable with uninterruptible supply systems (UPS) in order to meet the line needs. An uninterruptible power supply that consist of a lead-gel battery is also part of our system.

To control our system, we have used a control panel. It consists of a touchscreen with which the user can interact. It is fed from the 230 V mains, allows for updating via USB, and has password protection and remote-LAN programming. Furthermore, it includes the needed coupler for its integration in the system.

A KNX-IP gateway is necessary for providing external communication to the intermediate and advanced level, which allow for accessing the bus remotely. By this access, we have been able to carry out the programming of the system through a Virtual Private Network (VPN) configuration. System maintenance and diagnosis during full operation, and device management, have been carried out by means of InSideControl^®^ software. It requires a 24 V DC power supply and a modem/router Internet connection by RJ45 wire.

For line-main line connections or union of new areas with prior areas, we have installed line couplers. This device allows us to set up, according to some parameters, its behavior as a coupler or booster. This eases the effective range expansion of the bus. This also allows for void filter option to test the system. It has two connection terminals.

Finally, apart from the above-mentioned wire, connection terminals have been used to create branch lines or to connect some devices. To this aim, a product that allows the connection of four pairs with a single 0.6–0.8 diameter connection terminal was chosen.

In the following lines, we have described technical features of each implemented systems by means of tables and flow charts in order to ease the understanding of its function.

### 2.4. Data Acquisition (DAQ) System

A Data Acquisition (DAQ) system with several data loggers (MICROLITE 8/16), which allow us to capture lots of measures, has been implemented. These data loggers were integrated in all systems installed. The data were gathered every 20 min and once a month, there data are sent to a PC. Later, data are analyzed using several specialized software packages to obtain all the results presented in this paper.

## 3. Home Building Automation Systems

### 3.1. Basic Home Automation System

The basic system (home automation level 1) permits an automatic and efficient control over the lighting and air conditioning subsystem by means of several types of sensors and actuators, which are summarized in Table 1 and Table 2.

For the purpose of offering the user the possibility of managing the room’s temperature, a thermostat has been installed next to each bed. With it, the user can control the environment based on four stages, two hot/cold stages and two other auxiliary stages. In the event of voltage loss, the program saves itself and recovers automatically.

**Figure 1:**
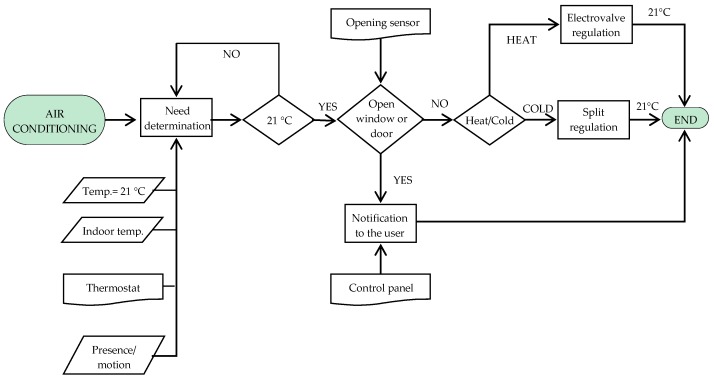
Air conditioning functioning flow chart.

**Figure 2:**
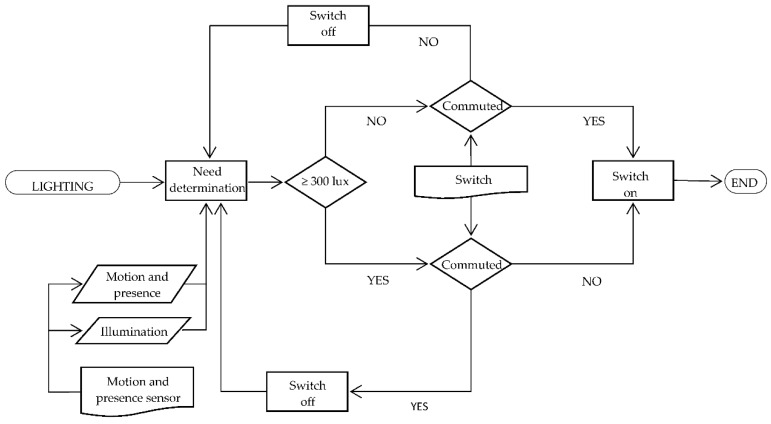
Lighting functioning flow chart.

In addition, we have installed a split control interface to establish a link between KNX system and air conditioners. It enables user control over devices by means of thermostats, avoiding remote control use. Figure 1 and Figure 2 are the air conditioning and lighting functioning flow charts, respectively.

Lastly, general features of our basic-level system are presented in Table 3.

**Figure 3:**
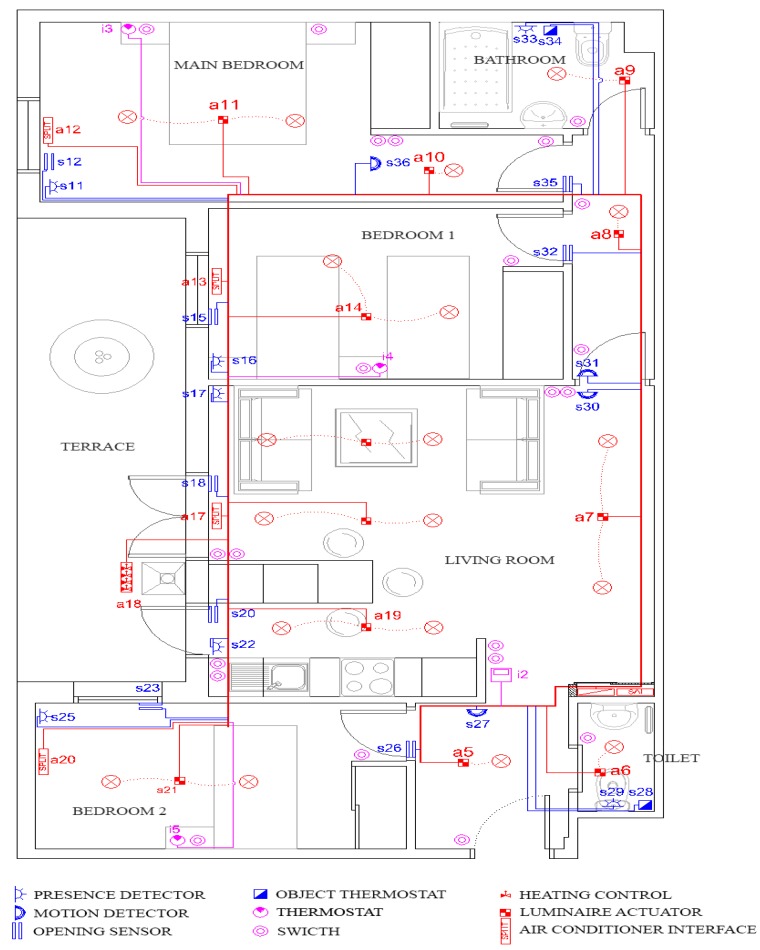
Components distribution for basic home automation system.

Because the number of devices is less than 64, it is possible to use only one line. However, the user will be limited if he wants to make enhancements. For this reason, we have installed lines in accordance with advanced system requirements. Our components (sensors, actuators, *etc.*) and line distribution all over the flat is reflected in Figure 3.

### 3.2. Intermediate Home Automation System

This system (home automation level 2) permits an automatic and efficient control over the lighting, air conditioning, water resources and the electrical power demand of the house. This system consists of the above-mentioned sensors and actuators in Table 1 plus the sensors and actuators in Table 4.

Five switches were also installed, each one situated next to each window. Finally, general features of our intermediate-level system are presented in Table 5.

### 3.3. Advanced Home Automation System

In this case, this system reaches a home automation level of 3. Apart from the functions that the intermediate system provides (automatic control over air conditioning, lighting, water resources and electrical power demand), it manages automatically dimmable lights and a multimedia system that is able to play sounds from several points of the flat. In addition, it can reproduce pre-recorded messages. What is more, it senses the presence and manages the dimmable lights situated in the terrace. Finally, it accurately senses the temperature gradient, and regulates the actuators, avoiding unpleasant currents. All of this is thanks to the sensors and actuators presented in Table 6.

To finish the description of the advanced home automation system, we present in Table 7 the general features of the system.

### 3.4. Technical Alarms Subsystem

This subsystem is attachable to the above-mentioned systems and it has the function of reporting to the user possible home disasters that might arise from facilities’ breakdowns such as fire, gas leakage or flooding. The installation of this subsystem means that an additional line and its respective area configuration, a power supply and an additional line coupler have to be added.

In order to avoid any unnecessary duplicity of elements, common elements between the systems and this subsystem are summarized below in Table 8.

Data are gathered from the sensors according to the table below (Table 9) and installed actuators are summarized in the same table.

**Figure 4:**
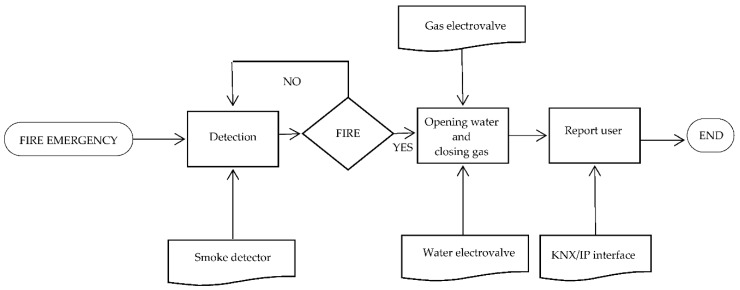
Flow chart of technical alarms subsystem functioning in case of fire emergency.

Then, the flow chart of the working of the subsystem in case of fire is shown in Figure 4. Table 10 presents the general features of this subsystem.

### 3.5. Security Subsystem

Similar to the technical alarm subsystem, the security subsystem is attachable to the above-mentioned home automation systems. Its main functions are to detect intrusions and potential failures that may ease them. It has direct external contact with the user and it can be installed in as an independent system. Its common elements with the main home automation systems are presented in Table 11.

**Figure 5:**
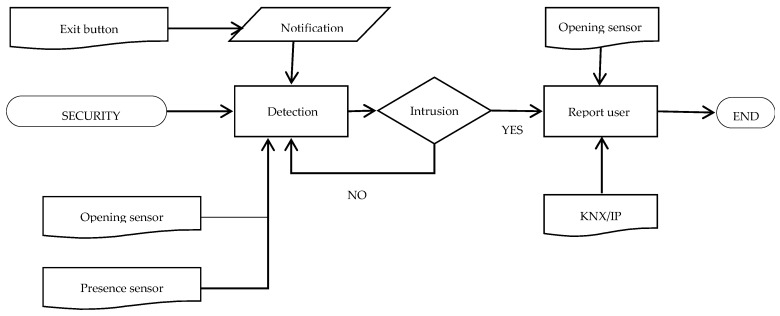
Security subsystem functioning flow chart.

Data gathered by the sensors (Table 12) are sent to a central security unit where the data are processed and, if needed, this central reports the user the information by means of InsideControl^®^. A button has also been installed to communicate with the subsystem an “Out of the house” or “In the house” state. A LED light changes color according to the state previously communicated. In Figure 5, a flow chart describes the working of this subsystem.

## 4. Results

After home automation systems implementation, several studies have been carried out, separated according to different variables.

In the following tables, the results are shown divided into subsections according to the same previous variables. These results are based on the coexistence of a family comprised of four people and the systems described.

### 4.1. Energy Study

The energy study found the following results, as shown in Table 13 and Table 14.

### 4.2. Economic Study

The economic study found the following results, as shown in Table 15 (implementation costs and economic savings) and Table 16 (payback periods).

### 4.3. Environmental Impact Study

We have studied CO_2_ emissions of the systems with the following results summarized in Table 17. In Table 18, percentages of savings depending on the areas in study and different locations [25] are summarized. These percentages have been obtained using SeeTool^®^ software introducing similar and real flats with the same orientation.

### 4.4. Non-Quantifiable Study

Apart from quantifiable variables, there are other system properties that the users appreciate when the system is implemented in their home. During the implementation phase and after the user–system coexistence phase, some questions were asked to the users.

Most of them, noted as the main benefit, the comfort the system provides to the user. The next most appreciated property was the remote control of their homes. 

Finally, but still important, the users emphasize the calm feeling from technical alarms and security subsystems.

## 5. Discussion

**Figure 6:**
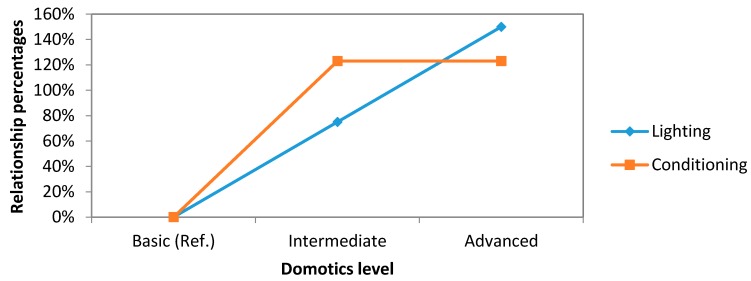
Comparative study graph concerning to energy savings (lighting and conditioning).

Two main comparisons have been carried out. The first one (Figure 6) compares the energy savings produced by the three domotics levels grouping lighting and conditioning consumptions.

**Figure 7:**
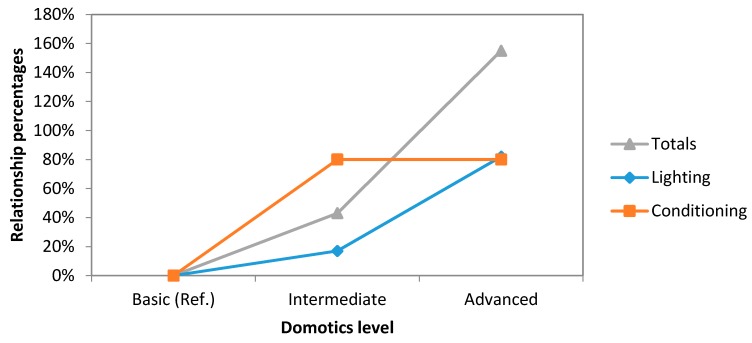
Comparative study graph concerning to economical savings (lighting and conditioning).

Furthermore, in the second (Figure 7), with the same groups, the relationship percentages between our systems has been compared.

Analyzing these two graphs, it can be noticed that the savings on lighting area have a linear trend while new improvements are made to our system, increasing the level of automation. However, this trend changes in economic area. There exists a higher increase in costs when the system levels up to advanced level from the intermediate one.

With regard to conditioning, its behavior is the same as the previous relation, no change existing between advanced and intermediate level but producing great savings. If we compare the intermediate system with respect to the basic one, the savings are improved by 123%.

As we can see in Figure 7, investments in installing the intermediate system only increase by 43% when the system levels up from basic system. However, this increase is multiplied by a factor of three if we compare it with the advanced system investment.

**Figure 8:**
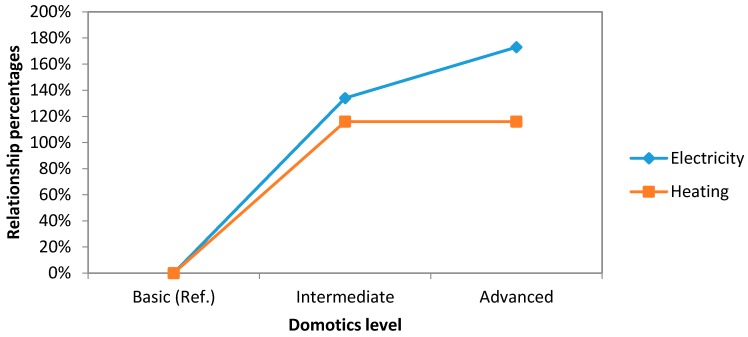
Comparative study graph concerning to energy savings (electricity and heating).

In Figure 8, the comparative graph concerning to energy savings of our three levels of automation related to electricity and heating areas can be found. Electricity area greatly increases on the passage from basic system to the intermediate one. This increase is stopped in intermediate-to-advanced step, reducing the relationship percentage of energy savings by 18%. Heating becomes stagnant at the same step.

**Figure 9:**
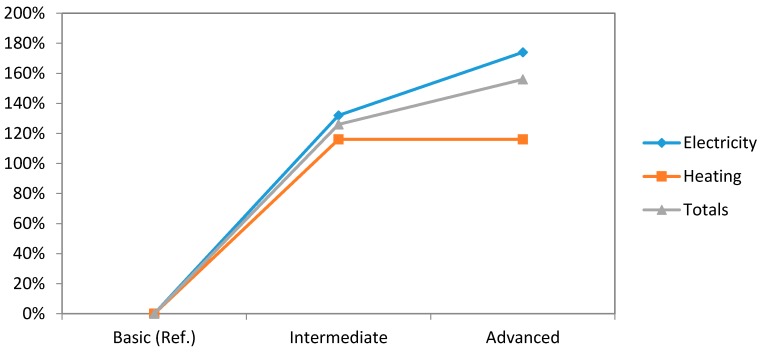
Comparative study graph concerning to CO_2_ emissions (electricity and heating).

If we compare these data and CO_2_ emissions (Figure 9), it is noticed that CO_2_ reduction comes from the savings done in electricity area and, especially, in the step from basic to intermediate level.

After we installed the home automation system, several surveys about its energy, economic and environmental impact have been done based on the four people who live in this flat. The results of these surveys show that our system achieves energy savings of 19.88% in lighting and 16.09% in air conditioning per year, as well as a reduction of CO_2_ emissions of 76.03 Kg/month (basic system). All of this implies economical savings of 300 €/year (basic system), and an amortization of the investment that has been made of 20 years (intermediate system).

It is important to notice that advanced system is not as good as expected. Its results are not much better than intermediate system results, being its implementation costs are 78% higher. Probably, its influence only in lighting area is not worthwhile. In the future, in order to get more economic savings, advanced system should take into consideration new tools in air conditioning area or some others that increase, significantly, these savings.

Apart from measurable factors, there are other not-measurable benefits that the user consider when implementing home automation systems. These are the acquired comfort and security feeling because of the security and technical alarms subsystems.

## 6. Conclusions

The implemented system in this dwelling offers a great number of objective advantages, having as its mainstay the energy savings, which also lead to economic savings and to a reduction of CO_2_ emissions, which is, nowadays, so valuable. It is also important to appreciate positively the increase in comfort and security that this system provides to the user.

Intermediate-to-advanced level up increases the costs of the system but there is little added economic savings; thus, the intermediate level is the most adequate for a small apartment typology. 

Climatic zones also influence on these savings; thus, apartment location is very important when choosing the best level of the home automation system.

The 20-year amortization, at best, is insufficient to conclude that the system is economically viable. This fact becomes a pending task that we will have to improve in the future to enter individual user market.

## Figures and Tables

**Table 1:** Installed sensors and actuators in basic home automation system.

Type	Number	Placement Criteria	Total Consumption (mA)	Comments
Opening sensor	8	1/window 1/airflow door	-	It requires KNX interface
Presence detector	7	1/room	56	Installation height = 2.20 m
Motion detector	4	1/passing area	16	It includes bus coupler and connection terminal
Temperature sensor	6	1/room	-	
Luminaires actuator	12	It depends on the luminaires number	75	Binary input to dry contacts connection or conventional push button
Heating electrovalve	6	1/room	5.2	
Split control interface	4	1/split	-	Bidirectional KNX gateway—Air conditioners

**Table 2:** Types and characteristics of sensors and actuators used.

Type	Comments
Opening sensor: KNX.CAGS142	When detecting the opening or closing of a window or door, a notification is sent to the control panel, which disables or activates air conditioning systems.
Presence detector: MTN630819	It covers an 8-m perimeter and is installed at a height of 2.2 m. It has a detection angle of 360 degrees.
Motion detector: MTN631719	It incorporates a light meter, a bus coupler and a terminal connection. It sends a signal to the control panel to turn the lights on or off depending on whether it detects the presence of someone or not.
Indoor temperature sensor: MTN6221-0319	It determines the temperature and sends it via bus to the logic module by activating the air conditioning until the temperature reaches 21 degrees (temperature setpoint). This activation also depends on the presence of users in the room.
Power Meter: MTN6600-0603	It can monitor up to three channels up to 3680 kW (total 3 × 3680 = 11,040 kW). Transmits data over the bus to the data logger. For its channels, it was reserved: one for air conditioning, one for lights and outlets and the last one for kitchen items.
External light sensor: MTN663991	Brightness and temperature are recorded by it. It communicates with the blind actuator, upping them if it exceeds 300 lux outside. Taking advantage of its temperature meter, it also communicates with heating and air conditioning to get the temperature to the setpoint (21 degrees).
Outdoor motion detector: MTN632519	It is prepared to be installed outside. Detecting the presence of people outside to control external lighting.
Temperature probes: MTN616790	They have been connected to up to four probes to a thermostat to improve the reading. It is installed at different points and at different heights to observe the homogenization temperature on the rooms.

**Table 3:** General features of basic home automation system.

**Number of Devices Connected to the Bus**	48	**Lines (Meters of Wire)**	1 (101)
**Combined Consumption (mA)**	168.20	**Power Supply**	1 × 320 mA
**Line Couplers**	0 (Only one line)	**External Communication**	No
**Control Device**	Control panel	**UPS**	No
**UPS Battery**	-	**Energy Self-Sufficiency**	-

**Table 4:** Installed sensors and actuators in basic home automation system.

Type	Number	Placement Criteria	Total Consumption (mA)	Comments
Electric meter	1	1/flat	12.5	Max. 11,040 W (3 × 3680 W)
Light and temperature sensor	1	1/terrace	6.25	Installation to measure light (specular reflection)
Binary-railed actuator for current measurement	1	1/kitchen	16	Binary input for connection to dry contacts or traditional switches
Blinds	5	1/window	17.5	
Automatic water recirculation system	1/3	1 impulsion module and 1 return module/wet area	8	Patent [24]

**Table 5:** General features of intermediate home automation system.

**Number of Devices Connected to the Bus**	60	**Lines (Meters of Wire)**	1 (101)
**Combined Consumption (mA)**	262.45	**Power Supply**	1 × 320 mA
**Line Couplers**	0 (Only one line)	**External Communication**	KNX/IP Gateway
**Control Device**	Control panel	**UPS**	No
**UPS Battery**	-	**Energy Self-Sufficiency**	-

**Table 6:** Installed sensors and actuators in advanced home automation system.

Type	Number	Placement Criteria	Total Consumption (mA)	Comments
Outdoors motion detector	1	1/terrace	13	
Temperature sensor	16	4/room	-	Two sensors at each measuring point, at heights and just above the floor
Binary actuator for lights	1	1/terrace	13	
KNX-DALI gateway	1	1/flat	5	Inside a cabinet
Electronic ballast	7	1/room with dimmable lights	0	Substitute the binary actuators
Background music/Communication system	1	1/flat	5	Includes iPod Nano to save pre-recorded messages

**Table 7:** General features of advanced home automation system.

**Number of Devices Connected to the Bus**	73	**Lines (Meters of Wire)**	2 (101)
**Combined Consumption (mA)**	277.45	**Power Supply**	1 × 640 mA 2 × 320 mA
**Line Couplers**	2	**External Communication**	KNX/IP Gateway
**Control Device**	Control panel	**UPS**	Yes
**UPS Battery**	7.80 Ah	**Energy Self-Sufficiency**	28 h

**Table 8:** Elements of technical alarms subsystem in common with the other systems.

	Basic	Intermediate	Advanced
KNX/IP interface	No	Yes	Yes
UPS Battery	No	No	Yes
Area power supply	No	No	Yes
Line couplers	No	No	Yes

**Table 9:** Installed sensors and actuators in advanced home automation system

Type	Number	Placement Criteria	Total Consumption (mA)	Comments
Flood sensor	1/3	1 central device and 1/wet area	65	
Optical smoke detector	6	1/bedroom, 1 over each sofa and 1/kitchen	2	
Gas detector for detection of methane	1	1 next to the boiler	132	
Binary electrovalve to water pipe	3	1/wet area	6	Supply from mains. It requires KNX interface
Binary electrovalve to gas pipeline	1	1 at the boiler	2	It requires KNX interface

**Table 10:** General features of advanced home automation system.

**Number of Devices Connected to the Bus**	18	**Lines (Meters of Wire)**	1 (45)
**Combined Consumption (mA)**	265.58	**Power Supply**	1 × 320 mA
**Line Couplers**	0	**External Communication**	KNX/IP Gateway
**Control Device**	Logic module	**UPS**	Yes
**UPS Battery**	7.80 Ah	**Energy Self-Sufficiency**	29 h

**Table 11:** Elements of security subsystem in common with the other systems.

	Basic	Intermediate	Advanced
KNX/IP interface	No	Yes	Yes
UPS Battery	No	No	Yes
Area power supply	No	No	Yes
Line couplers	No	No	Yes
Opening sensor	Yes	Yes	Yes
Presence sensor	Yes	Yes	Yes
Exit button	No	Yes	Yes

**Table 12:** Elements of security subsystem in common with the other systems.

Type	Number	Placement Criteria	Total Consumption (mA)	Comments
Opening sensor	5	1/outer door or outer window	0	
Break sensor	5	1/outer door or outer window	50	
Presence sensor	5	1/outer room	0	Synergy with basic system

**Table 13:** Lighting and air conditioning energy study results (monthly).

	System	Main features	Resource Savings		% Energy Savings	
**Lighting Management (kWh)**	Basic	- Presence control - Light control	6.80		19.88	
	Intermediate	- All the previous ones - Optimization solar system	11.90		34.79	
	Advanced	- All the previous ones - Dimming	17.01		50	
			**Heating**	**Air Conditioning**	**Heating**	**Air Conditioning**
**Air Conditioning Management (kWh)**	Basic	- Control by presence - Schedule programming	147.02	64.58	17.82	14.35
	Intermediate	- All the previous ones - Optimization solar system(blinds)	317.05	154.17	38.43	34.26
	Advanced	- All the previous ones	317.05	154.17	38.43	34.26

**Table 14:** Water resources energy study results.

	System	Main Features	Resource Savings (L/Day)	% Energy Savings
**Water Resources Management**	SARAP [24]	Automatic water recirculation system	180	33.84

**Table 15:** Implementation costs and economic savings results (€).

System	Total	Lighting	Air Conditioning	Power Term	Water Resources	Monthly Savings	Annual Savings
**Basic**	9613.86	1.25	23.78	-	-	25.03	300.40
**Intermediate**	13,737.46	1.46	42.72	4.56	7.40	56.14	673.68
**Advanced**	24,533.66	2.28	42.72	4.56	7.40	56.96	683.52
**Technical Alarms**	3871.83	-	-		-	-	-
**Security**	3548.31	-	-		-	-	-

**Table 16:** Payback period results.

	Basic	Intermediate	Advanced
**Payback Period (Years and Months)**	32 years	20 years and 5 months	38 years and 4 months

**Table 17:** CO_2_ emissions results.

	System	Conversion Factor (gr CO_2_/kWh)	Energy Savings (kWh)	Reduction of CO_2_ Emissions (kg/Month)
Electricity	Basic	645	71.38	46.04
	Intermediate		166.07	107.12
	Advanced		195.4	126.03
Gas	Basic	204	147.02	29.99
	Intermediate		317.05	64.68
	Advanced		339.32	69.22

**Table 18:** Percentages of savings in lighting depending on its location.

	System	Madrid (%) (Continental Zone)	Barcelona (%) (Mediterranean Zone)	A Coruña (%) (Atlantic Zone)
Lighting management	Basic	19.88	51.49	27.43
	Intermediate	34.79	70.11	48.01
	Advanced	50.00	85.50	69.01
Heating	Basic	17.82	9.91	9.02
	Intermediate	38.43	21.35	19.41
	Advanced	38.43	21.35	19.41
Air conditioning	Basic	14.35	15.78	5.35
	Intermediate	34.26	37.69	12.77
	Advanced	34.26	37.69	12.77
Water resources	Basic	33.84	30.21	31.05
	Intermediate			
	Advanced			

## Abbreviations

HBAS	Home Building Automation System
BAS	Building Automation System
IPTV	Internet Protocol Television
API	Application Programming Interface
KNX	Konnex
CSMA/CA	Carrier sense multiple access with collision avoidance
EPROM	Erasable Programmable Read-Only Memory
ROM	Read-Only Memory
RAM	Random Access Memory
VPN	Virtual Private Network
DAQ	Data Acquisition
UPS	Uninterruptible power supply
